# Anatomical alignment of the acetabular component using transverse acetabular ligament in total hip replacement: a prospective cohort study

**DOI:** 10.1038/s41598-025-19846-2

**Published:** 2025-10-14

**Authors:** Dinh-Hieu Nguyen, Trung-Tuyen Nguyen, Van-Hieu Dang, Ba-Hai Nguyen, Khanh-Trinh Le, Son-Tung Pham, Ngoc-Hoang Bui, Van-Nam Le, Duc-Nam Vu, Trung-Dung Tran, Hoang-Long Vo

**Affiliations:** 1 Department of Orthopaedic Surgery and Sports Medicine, E Hospital, Hanoi, 100000 Vietnam; 2 University of Medicine and Pharmacy, Vietnam National University, Hanoi, 100000 Vietnam; 3 Hanoi Medical University, Hanoi, 100000 Vietnam; 4 Department of Orthopaedic Surgery, College of Health Science, VinUniversity, Hanoi, 100000 Vietnam; 5 Orthopaedic and Sports Medicine Center, Vinmec Healthcare System, Hanoi, 100000 Vietnam; 6 Department of Science, Technology, Communication & International Cooperation, E Hospital, Hanoi, 100000 Vietnam

**Keywords:** Anatomical alignment, Anatomical landmark, Transverse acetabular ligament, Total hip replacement, Anatomy, Outcomes research

## Abstract

Optimal positioning of the acetabular component is crucial in total hip replacement surgery to minimize postoperative dislocation rates. The transverse acetabular ligament (TAL) has been proposed as a useful anatomical landmark for cup orientation. This prospective cohort study included 122 patients who underwent total hip replacement at E Hospital, Hanoi, between January 2021 and December 2022. Orientation angles of the TAL and acetabulum were assessed preoperatively using CT with multiplanar reconstruction and MRI arthrography, and intraoperatively with a handmade protractor developed at our institution. The mean anteversion of TAL on CT and MRI was approximately 10°, with a mean inclination of 45–46°. Intraoperatively, the mean TAL anteversion was 10.2° and the acetabular anteversion was 12.0°, while the mean TAL inclination was 44.9° and the acetabular inclination 41.9°. These findings demonstrate significant correlations between TAL orientation and acetabular alignment across imaging and intraoperative measurements. TAL is a readily identifiable landmark, and its use can facilitate accurate, patient-specific acetabular cup positioning within the safe zone, thereby enhancing surgical outcomes.

## Introduction

Total hip replacement, widely regarded as one of the most successful surgeries of the 21 st century, annually aids millions of patients by treating and repairing damaged hip joints due to many different causes. The most common indications for total hip replacement include hip fractures, osteoarthritis, and osteonecrosis of the femoral head.

Proper placement of the acetabular component in total hip replacement enhances surgical outcomes by increasing the stability of the artificial joint, reducing impingement, minimizing wear on the artificial joint, and avoiding the risk of dislocation. Most surgeons today tend to achieve the cup position within Lewinnek’s “safe zone”^1^ including the inclination from 30^o^ to 50^o^, and the version from 5^o^ to 25^o^. Anatomical referencing is a method of using the patient’s existing anatomical structures as a landmark to place the acetabulum component. There are many anatomical structures used such as the native acetabular, anterior and posterior horn of the acetabular notch, acetabular rim cartilage system, and most notably, the transverse acetabular ligament (TAL), which is recommended by many authors. Archbold found that when the acetabular component was placed parallel to the TAL, the postoperative dislocation rate was 0.6%^2^. Revanngowda’s research indicated that using the TAL as an anatomical landmark resulted in 96.15% of the version angles and 84.6% of the inclination angles falling within the Lewinnek’s safe zone^3^.

This study sought to demonstrate the directional correlation between TAL and the acetabulum based on preoperative multislice computed tomographic system with multiplanar reconstruction (CT-MPR) and MRI arthrography (MR-a). With preoperative planning using CT-MPR and MR-a, we can determine the orientation angles of the TAL and the acetabulum, compare with intraoperative parameters based on a handmade protractor to determine the linear correlation, and subsequently evaluate the role of the TAL as an anatomical landmark.

## Materials and methods

### Study design & patients

From January 2021 to December 2022, we prospectively analyzed a total of 122 acetabula in the patients who received indications for total hip arthroplasty at our institution (Department of Orthopaedic Surgery and Sports Medicine of the E Hospital, Hanoi, Vietnam). Inclusion criteria were: (1) adult patients (≥ 18 years) who underwent primary total hip arthroplasty (THA) at E Hospital between 1 January 2021 and 31 December 2022; (2) primary surgical indications of femoral neck fracture, primary osteoarthritis, or avascular necrosis of the femoral head; (3) availability of preoperative pelvic CT with multiplanar reconstruction (CT-MPR); (4) intraoperative measurement of TAL and acetabular orientation using the handmade protractor developed at our institution; and (5) written informed consent for study participation and for the imaging procedures. Exclusion criteria were: (1) prior ipsilateral hip surgery (including internal fixation, osteotomy, or previous arthroplasty); (2) active hip joint or systemic infection; (3) pelvic or proximal femoral tumor; (4) severe congenital or acquired acetabular deformity or marked bone loss that precluded reliable identification of the transverse acetabular ligament (TAL); (5) contraindication to MR-arthrography (for example, MRI-incompatible implant, severe renal impairment, allergy to contrast) or patient refusal to undergo MR-arthrography; and (6) incomplete preoperative imaging or inability to obtain intraoperative measurements because the TAL could not be identified. Each hip was analyzed as a separate unit; bilateral procedures were counted as separate hips. Note on MR-arthrography: MR-arthrography was planned for all patients as part of preoperative assessment; however, MR-a was not performed in cases with contraindications or patient refusal. Therefore, MR-a measurements are reported for the subset of hips for which MR-a was available (*n* = 109).

Preoperative characteristics of the patients were, in detail, described in Table 1. The study patients consisted of 84 males (68.85%) and 38 females (31.15%). The mean age was 59.25 ± 13.15 years (range: 24–87). The mean BMI was 22.17 ± 2.84 kg/m² (range: 11.66–31.64). Indications for surgery included hip fracture in 57 patients (46.72%), osteoarthritis in 25 patients (20.49%), and osteonecrosis in 40 patients (32.79%).

**Table 1:** Preoperative characteristics of the patients.

Characteristics	Patients (*N* = 122)
Age	
Mean ± SD, years	59,25 ± 12,41
Range, years	24–87
≥ 60 years, no. (%)	59 (48.36)
Female sex, no. (%)	38 (31.15)
Reasons for hospitalization, no. (%)	
Right groin pain	48 (39.34)
Left groin pain	57 (46.72)
Bilateral groin pain	17 (13.93)
Time from admission to surgery	
Mean ± SD, days	2,88 ± 3,15
Range, days	0–18
Preoperative diagnosis	
Fracture	57 (46.72)
Osteoarthritis	25 (20.49)
Avascular necrosis	40 (32.79)
BMI (kg/m^2^)	
Mean ± SD, kg/m^2^	22.17 ± 2.84
Range, kg/m^2^	11.66–31.64

BMI: body mass index; SD: standard deviation.

### Preoperative measurement

**Fig. 1:**
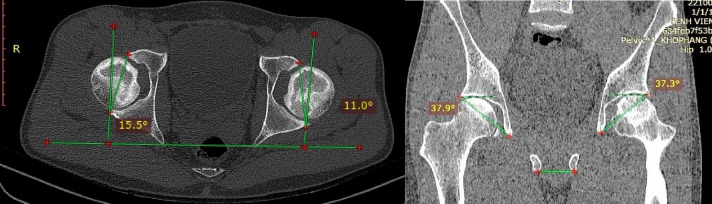
Preoperative CT-MPR-based measurement of acetabular anteversion angle and acetabular inclination angle^4^.

**Fig. 2:**
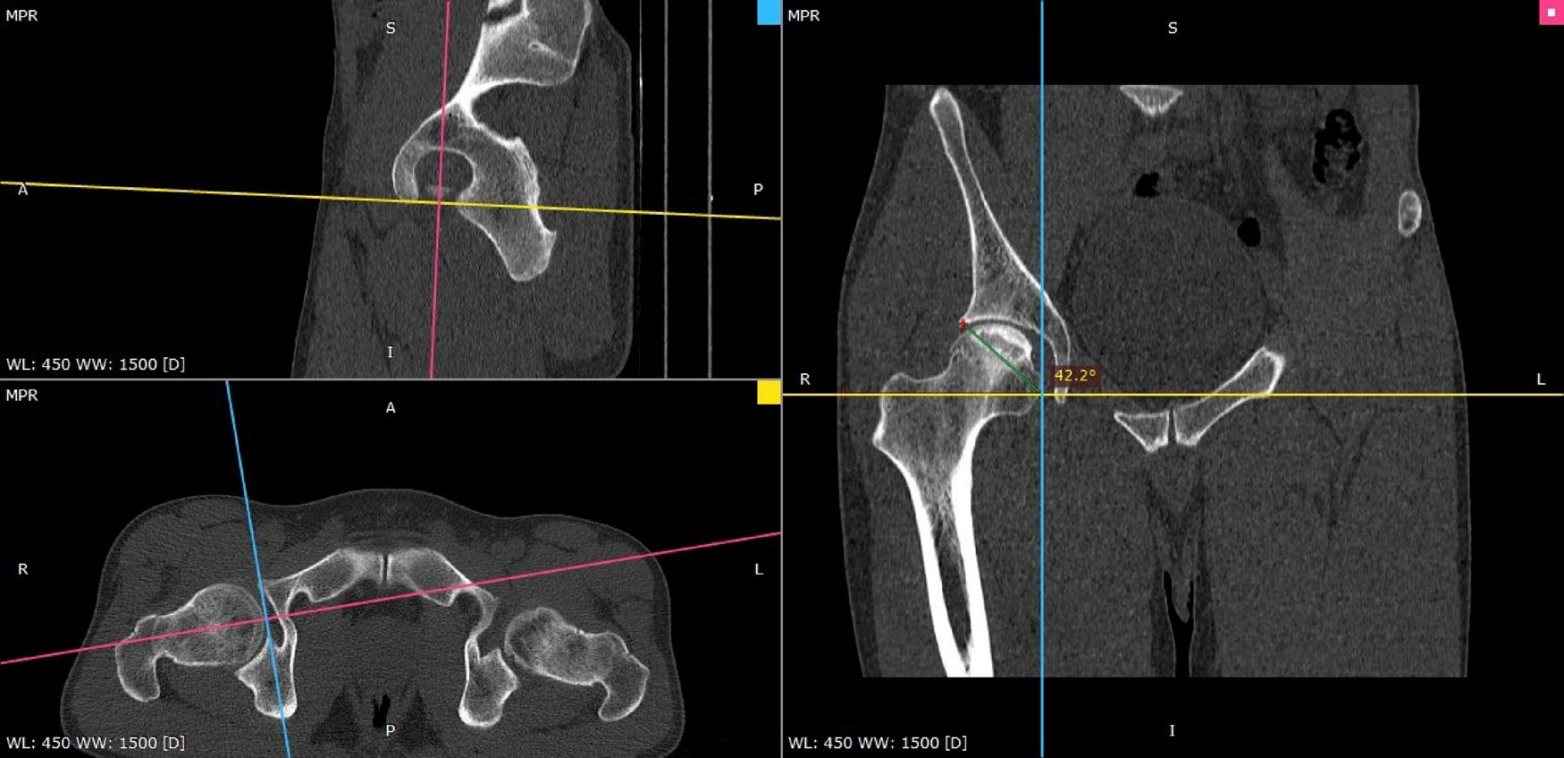
Preoperative determination of TAL and measurement of TAL inclination angle by CT-MPR^4^.

The 64 Slice Siemens SOMATOM Perspective CT Scanner with model DC045D from Germany for imaging was applied for preoperatively measuring orientation angles of the TAL and the acetabulum. Patients were scanned in a supine position and balanced on the scanner table, with their legs in a neutral position and their knees extended straight at 0 degrees. Imaging parameters: the field of view (FOV) width ranges from 300 to 400 mm; the voltage is between 110 and 140 kV; the current intensity is 100 mAs; the slice thickness is 0.6–1 mm; the reconstruction interval is 1–1.2 mm for the bone window and the software on the coronal, axial, and sagittal planes; and the table feed is 0.8–1.2 mm. Then the orientation angle of the acetabulum on the axial and coronal planes were measured by CT-MPR (Fig. 1). We also determined and measured the TAL orientation on CT-MPR (Fig. 2).

**Fig. 3:**
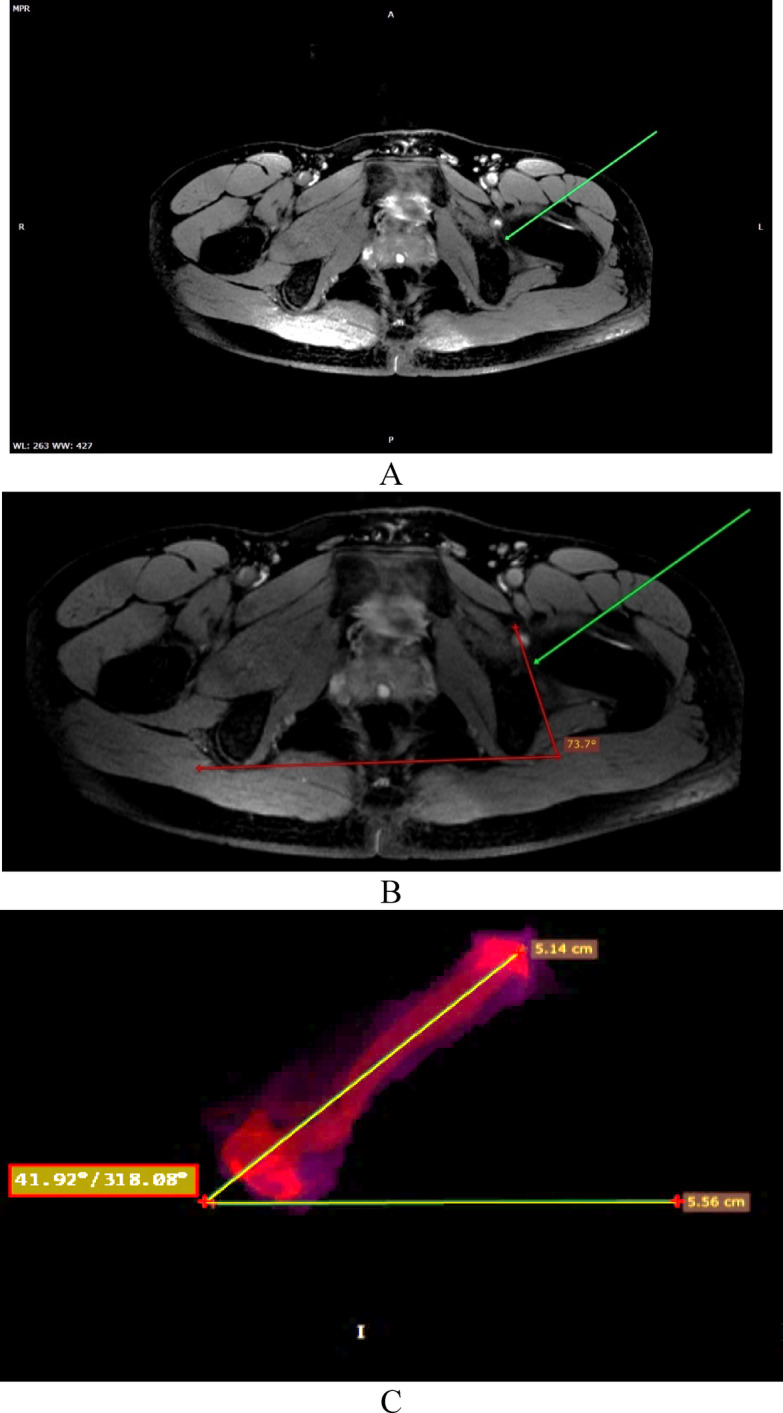
Preoperative determination of TAL **(A)** and measurement of the angle of TAL anteversion angle **(B)** and TAL inclination angle **(C)**.

MR-a of the hip joint was performed using a 2-step procedure^5^. Step 1: Inject contrast. The patient Lies on a slightly elevated table with a pillow placed under the kneecap. The hip joint is rotated 15 degrees by securing the big toes together with adhesive tape. Inject 10 ml of magnetic contrast through a 20G lumbar puncture needle. Step 2: Perform MR-a with T1-weighted fat-saturation pulse sequence (TR = 600 ms/TE = 14 ms) on 3 planes. T2-weighted fat-saturation pulse sequence (TR = 3000ms/TE = 90ms) images are obtained in the coronal plane. All sequences on the diagonal axis are imaged with a 5 mm slice thickness, FOV 24, and a 320 × 224 image matrix. On the sagittal plane, the image matrix is 256 × 224 (Fig. 3).

### Intraoperative measurement

**Fig. 4:**
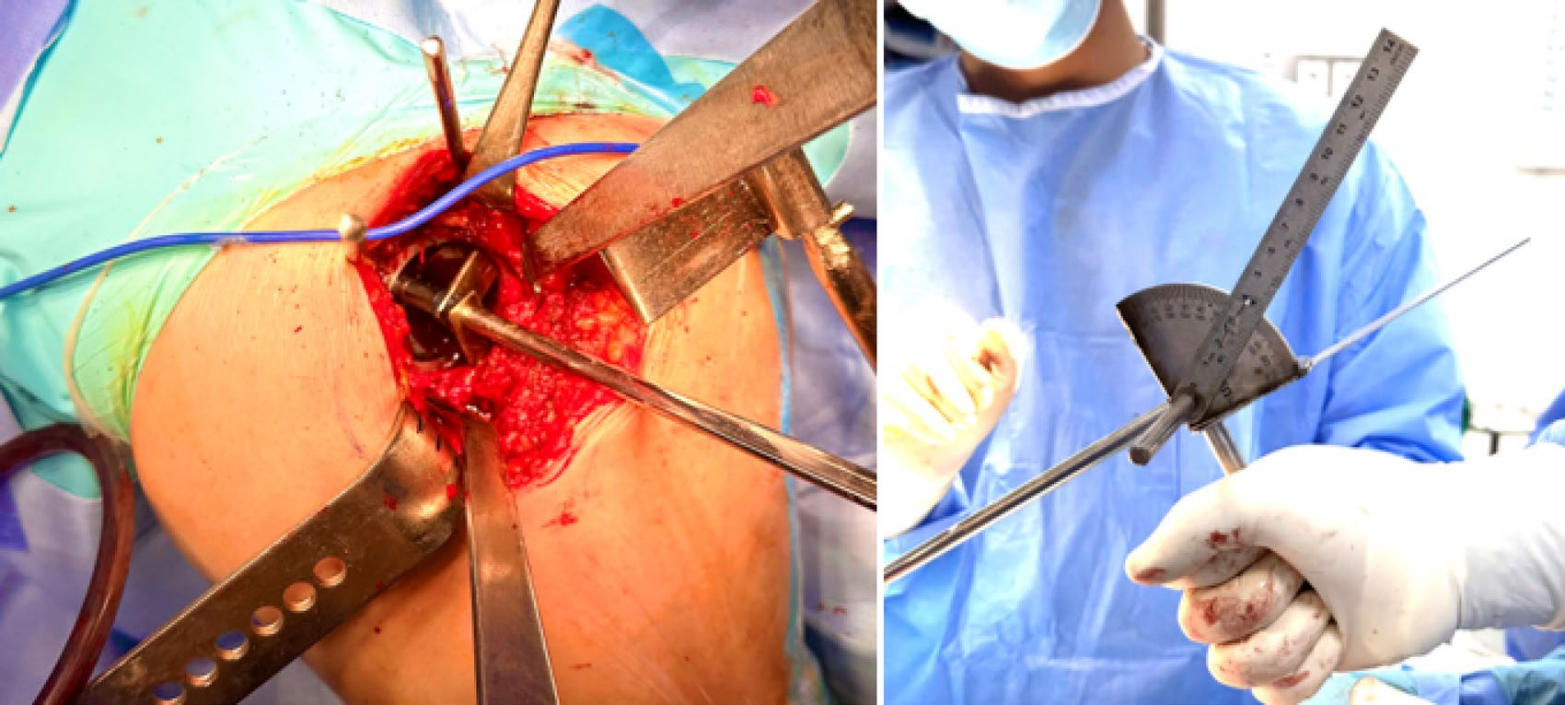
Intraoperative determination of TAL and and acetabulum and measurement of orientation angle of TAL and acetabulum by handmade protractor.

**Fig. 5:**
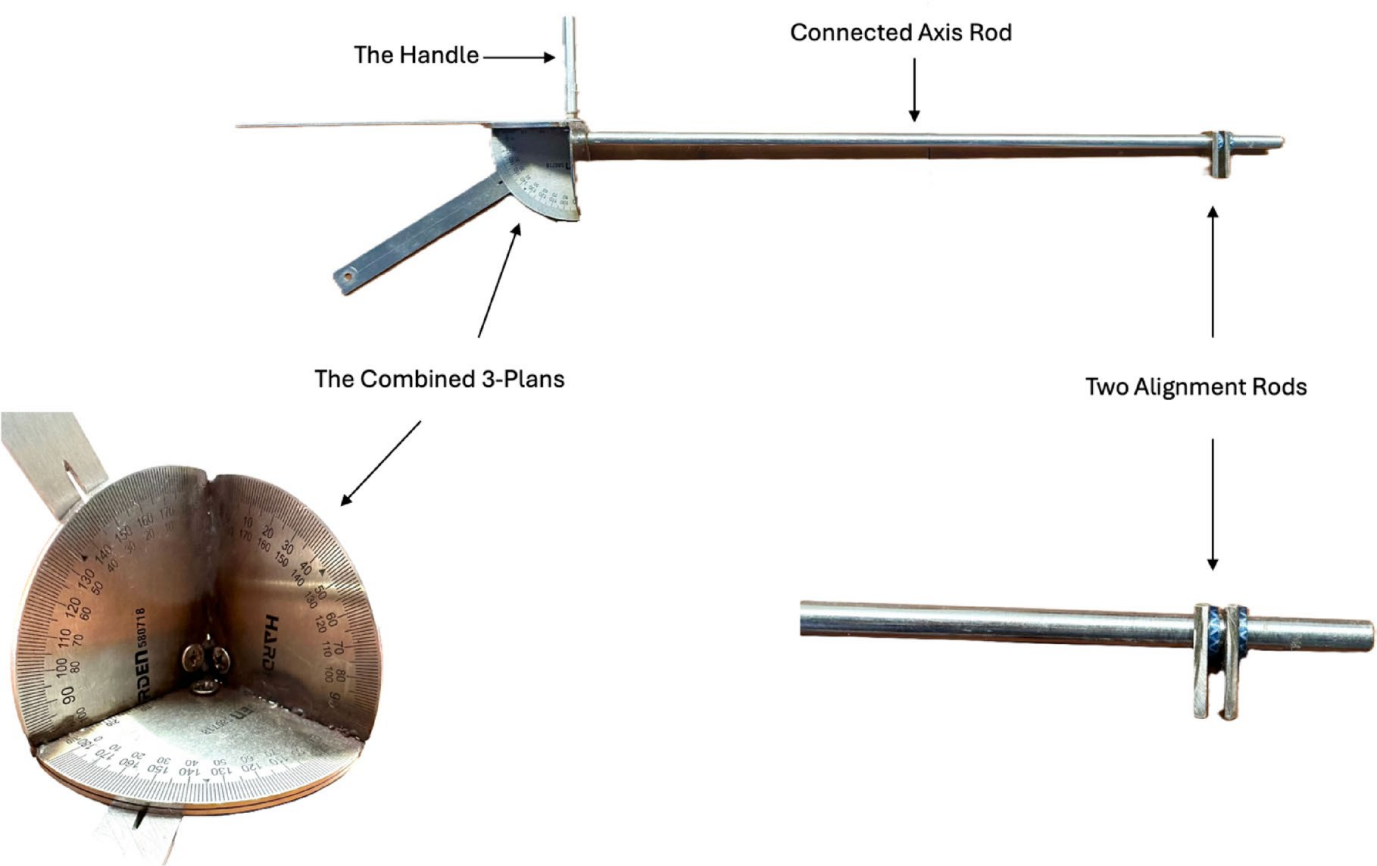
Structure of the handmade protractor developed by E Hospital for intraoperative measurement.

Intraoperatively, we used a handmade protractor with 2 alignment rods placed into the acetabulum and TAL to measure the orientation angle of these two structures (Fig. 4). This handmade protractor is developed by our institution (E Hospital) and is being introduced for the first time in this study. The protractor’s structure is created by combining a system of 3 planes and 2 alignment rods. The position of the two alignment rods can be adjusted to coincide with the plane to be measured. The entire protractor system is mounted on a handle for easy use during surgery (Fig. 5).

All surgeries were performed using the direct lateral approach (Hardinge approach), with patients positioned in the lateral decubitus position on a standard operating table.

### Statistical analysis

Parameters were measured using RadiAnt DICOM Viewer software. The data obtained was entered into EpiData 3.1, and responses were coded appropriately before being exported to Stata^®^ 15 (StataCorp LLC, College Station, TX, USA) for analysis. All data was first performed a visual inspection for coding errors, outliers, or funky distributions. We tabulated the proportion of patients with the baseline characteristic variables listed above for those received indications for total hip arthroplasty, using means and standard deviation for continuous variables and frequency and proportions for categorical variables. Preoperative and intraoperative anatomical parameters of acetabulum and TAL were presented using means and standard deviation. The orientation angles of the TAL and the acetabulum between two genders were compared using either the Mann-Whitney U test or the t-test. The Pearson and Spearman coefficients (r) were utilized to evaluate the correlation between orientation angles of the acetabulum and the TAL. *p*-value < 0.05 was considered to be statistically significant.

## Results

The analysis included a total of 122 acetabula. As was shown in Table 2, there were no significant difference in the preoperative orientation angles of the TAL and the acetabulum on CT-MPR between men and women.

**Table 2:** Preoperative measurements for the orientation angles of the TAL and the acetabulum on CT-MPR.

	All acetabula (*N* = 122)		Male (*N* = 84)		Female (*N* = 38)		*p*-values
	Mean (SD)	Min-max	Mean (SD)	Min-max	Mean (SD)	Min-max	
cAIA (degree)	41.86 (5.43)	30–67	41.46 (4.75)	30-60.1	42.76 (6.72)	30–67	0.6225^M^
cAAA (degree)	12.24 (4.96)	2.5–28.9	11.60 (3.80)	4.9–20.1	13.68 (6.75)	2.5–28.9	0.1786^M^
cAAS (degree)	38.93 (5.81)	210 − 51.2	38.51 (4.24)	25- 45.7	39.88 (8.35)	21.5–51.2	0.0525^M^
cSAA (degree)	24.30 (10.10)	3.7–48.7	24.18 (9.58)	4.2–43.9	24.57 (11.31)	3.7–48.7	0.5756^T^
cTIA (degree)	46.17 (8.10)	31.8–89	45.86 (7.18)	35.3–87	46.87 (9.947)	31.8–89	0.5814^M^
cTAA (degree)	9.94 (3.81)	2.5–18.7	9.89 (3.74)	3.1–18.7	10.04 (4.02)	2.5–18.6	0.5808^T^

cAIA: acetabular inclination angle; cAAA: acetabular anteversion angle; cAAS: acetabular angle of sharp; cSAA: sagittal acetabular angle; cTIA: TAL inclination; cTAA: TAL anteversion; SD: standard deviation.

M = Mann–Whitney U test; T = ttest.

Preoperative correlations amongst the orientation angles of the TAL and the acetabulum on CT-MPR were shown in Table 3. Significant positive correlations were observed between the inclination of the TAL and the acetabulum (*p* < 0.05; *r* = 0.4207), and between the anteversion of the TAL and the acetabulum (*p* < 0.05; *r* = 0.4107).

**Table 3:** Preoperative correlations amongst the orientation angles of the TAL and the acetabulum on CT-MPR.

	cAIA	cAAA	cAAS	cSAA	cTIA	cTAA
cAIA	1					
cAAA	0.0199	1				
cAAS	0.3796*	0.0893	1			
cSAA	0.3319*	0.2522*	0.3480*	1		
cTIA	0.4207*	0.0060	0.2892*	0.2651*	1	
cTAA	0.0100	0.4107*	−0.0480	0.0612	−0.1528	1

cAIA: acetabular inclination angle; cAAA: acetabular anteversion angle; cAAS: acetabular angle of sharp; cSAA: sagittal acetabular angle; cTIA: TAL inclination; cTAA: TAL anteversion.

*Significance level at *p* < 0.05.

Table 4 showed there were no significant difference in preoperative measurements for the orientation angles of TAL on MR-a between men and women.

**Table 4:** Preoperative measurements for the orientation angles of TAL on MR-a.

	All acetabula (*N* = 109)		Male (*N* = 77)		Female (*N* = 32)		*p*
	Mean (SD)	Min-max	Mean (SD)	Min-max	Mean (SD)	Min-max	
mTIA (degree)	45.18 (5.58)	33.5–57.5	45.10 (5.28)	33.5–55.9	45.39 (6.33)	33.8–57.5	0.8443^M^
mTAA (degree)	9.89 (3.80)	3.2–18.1	9.70 (3.62)	3.2–17.2	10.35 (4.21)	3.2–18.1	0.7883^T^

mAIA: acetabular inclination angle, mAAA: acetabular anteversion angle, mTIA: TAL inclination, mTAA: TAL anteversion; SD: standard deviation.

M = Mann–Whitney U test; T = ttest.

Table 5 indicated there were significant positive correlation between cTIA and mTIA (*p* < 0.05; *r* = 0.8656), and between cTAA and mTAA (*p* < 0.05; *r* = 0.9555).

**Table 5:** Preoperative correlations amongst the orientation angles of the TAL on CT-MPR and MR-a.

		MR-a	
		mTIA	mTAA
CT-MPR	cTIA	0.8656^S^*	−0.1778^S^
	cTAA	−0.3399^S^*	0.9555^P^*

cAIA: acetabular inclination angle on CT-MPR, cAAA: acetabular anteversion angle on CT-MPR, mTIA: TAL inclination on MR-a, mTAA: TAL anteversion on MR-a.

*significance level at *p* < 0.05.

Table 6 showewd there was no significant difference between men and women according to the angles describing the orientation of the acetabulum and TAL intraoperative.

**Table 6:** Intraoperative measurements for the orientation angles of the TAL and the acetabulum.

	All acetabula (*N* = 122)		Male (*N* = 84)		Female (*N* = 38)		*p*-values
	Mean (SD)	Min-max	Mean (SD)	Min-max	Mean (SD)	Min-max	
oAAA (degree)	11.97 (4.87)	3.1–39.2	11.69 (4.53)	5-39.2	12.60 (5.57)	3.1–25.1	0.2799^M^
oAIA (degree)	41.87 (4.88)	30–60	41.52 (4.34)	30-55.8	42.68 (5.92)	30.3–60	0.4388^M^
oTIA (degree)	44.85 (5.45)	32.3–58.7	44.78 (4.99)	35.5–57.7	45.03 (6.44)	32.3–58.7	0.5909^T^
oTAA (degree)	10.24 (3.52)	3.2–19.2	10.08 (3.41)	3.3–17.3	10.61 (3.79)	3.2–19.2	0.7743^T^

oAAA: acetabular anteversion angle intraoperative, oAIA: acetabular inclination angle intraoperative, oTIA: TAL inclination intraoperative, oTAA: TAL anteversion intraoperative.

M = Mann–Whitney U test; t = ttest.

Table 7 indicated there were significant positive correlations of oAAA with cAAA(*r* = 0.9257) and cTAA (*r* = 0.5160), of oAIA with cAIA (*r* = 0.9830) and cTIA (*r* = 0.4079), of oTIA with cTIA (*r* = 0.9006) and cAIA (*r* = 0.4008), and of oTAA with cTAA (*r* = 0.9350) and cAAA (*r* = 0.4839).

**Table 7:** Correlations of orientation angles of acetabulum and TAL between preoperative CT-MPR and intraoperative measurement.

		Intraoperative			
		oAAA	oAIA	oTIA	oTAA
Preoperative CT-MPR	cAAA	0.9257*	0.0527	−0.0053	0.4839*
	cAIA	−0.0545	0.9830*	0.4008*	0.0004
	cTIA	−0.0286	0.4079*	0.9006*	−0.1723
	cTAA	0.5160*	−0.0000	−0.2097*	0.9350*

cAAA: acetabular anteversion angle on CT-MPR, cAIA: acetabular inclination angle on CT-MPR, cTIA: TAL inclination on CT-MPR, cTAA: TAL anteversion on CT-MPR, oAAA: acetabular anteversion angle intraoperative, oAIA: acetabular inclination angle intraoperative, oTIA: TAL inclination intraoperative, oTAA: TAL anteversion intraoperative.

*significance level at *p* < 0.05.

As was shown in Table 8, there were significant correlations amongst angle orientation parameters of TAL between preoperative MR-a and intraoperative measurement.

**Table 8:** Correlations amongst angle orientation parameters of TAL between preoperative MR-a and intraoperative measurement.

		Intraoperative	
		oTIA	oTAA
Preoperative MR-a	mTIA	0.9406*	−0.3422*
	mTAA	−0.2413*	0.9607*

mTIA: TAL inclination on MR-a, mTAA: TAL anteversion on MR-a, oTIA: TAL inclination intraoperative, oTAA: TAL anteversion intraoperative.

*significance level at *p* < 0.05.

## Discussion

Lewinnek’s safe zone is defined as an acetabular inclination angle between 30° and 50° and an acetabular anteversion angle between 5° and 25°^1^. In total hip replacement surgery, positioning the acetabular component within the Lewinnek’s safe zone significantly reduces the rates of postoperative dislocation, impingement, and tendinopathy. Many authors recommend using the TAL as an anatomical landmark, citing a high success rate in achieving the safe zone postoperatively^3^.

**Fig. 6:**
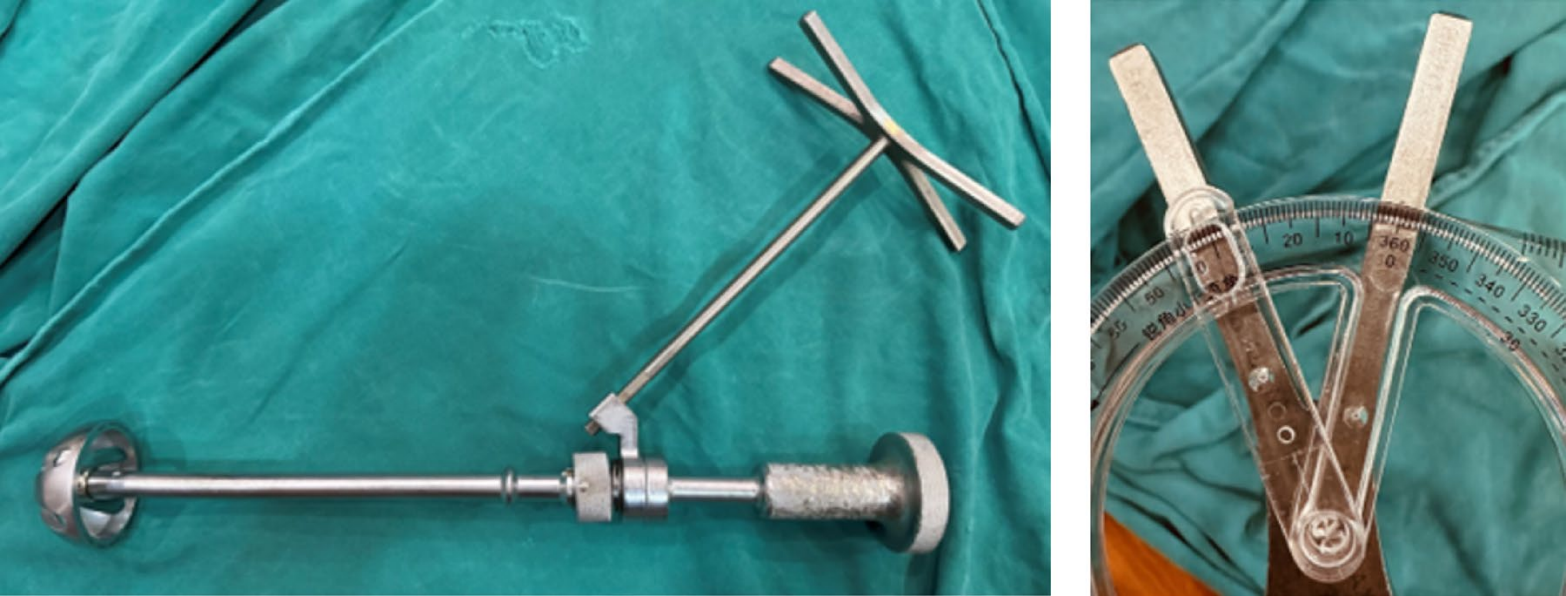
Traditional alignment instrument commonly used in Vietnam.

In Vietnam, options for aligning the acetabular cup orientation are currently Limited. The most commonly used tool is the traditional alignment instrument with fixed angles, lacking individual customization. This instrument typically has a fixed inclination angle of 45° and a fixed version angle of 20° (Fig. 6). Consequently, accurate axis alignment still heavily depends on the surgeon’s experience.

To increase customization and allow intraoperative measurement of acetabular and TAL orientation angles, our institution have designed a protractor system. The basic principle involves aligning the structural plane to be measured within the surgical field with a parallel plane outside the surgical field. This allows the orientation angle of that plane to be measured using a protractor system that forms three planes perpendicular to the Ox, Oy, and Oz axes. Each plane has a protractor engraved for easy observation. This simple protractor can be easily applied in surgery to measure the orientation angles of the acetabulum and the TAL, and to guide the reaming angle of the acetabulum and the placement of the acetabular cup. However, the disadvantage of this protractor is that its accuracy is not as high as electronic protractors, and its numerous small components make cleaning and sterilization challenging. We will continue to optimize this system in subsequent studies. To address the limitations of the handmade protractor, such as its inability to account for intraoperative pelvic motion and its lower accuracy compared to electronic systems, integrating real-time pelvic tracking systems or optical navigation in future iterations of the device is a potential solution. These technologies could provide dynamic feedback on pelvic orientation during surgery, enhancing the precision of cup positioning and reducing variability. By combining these advancements with the simplicity and cost-effectiveness of our current protractor design, we aim to create a more robust and reliable tool for acetabular alignment.

Our study results showed that there was a positive correlation in the inclination angle and anteversion angle between TAL and the acetabulum when evaluated on CT-MPR and MR-a, as well as with the orientation angle measured intraoperatively. A series of previous studies evaluating the role of TAL in orient cup position were presented in Table 9. Archolbd whose first study results were reported in 2006^2^. Regarding the role of TAL in orienting the anteversion of the cup component, 11 out of 19 studies recommended that TAL was a useful landmark for cup positioning in terms of anteversion. For the inclination angle, 8 out of 11 studies conclude that TAL can be used to determine cup inclination.

**Table 9:** Literature review on the role of TAL in cup Positioning.

No.	Author (year)	Number of acetabula (M/F)	Method	Conclusion
1	Archbold (2006)^2^	1000 (463/537)	Intraoperative oriented cup component parallel TAL	The ratio dislocation post-op was 0.6%.
2	Archbold (2008)^6^	25 (NA)	Measured inclination and anteversion of TAL on MR-a and intraoperative and assessment correlation	The TAL offers a possible solution to cup placement in THA
3	Epstein (2011)^7^	64 (NA)	Comparative between 2 groups: TAL definied (30) and TAL indefinied (34). Using TAL as an anatomy landmark to cup placement when defined.	TAL can be defined in approximately 30–60%, using TAL guide the cup orientation, the radiographic are comparable but not better than traditional guide instruments.
4	Kalteis (2011)^8^	40 (15/25)	Using navigation intraoperative to measure anatomy landmarks as well as for definitive placement of component	Alignment of acetabular component relative to the TAL landmark can reduce malpositioning acetabular component in THR according to the traditional safe zone.
5	Viste (2011)^9^	8 (3/5) with 14 acetabula	Using motion analysis in 14 cadaveric pelves normal acetabula. Measure anteversion of TAL-labrum	The TAL seems to be specific reference for each patient but its reliability must still be confirmed as an adequate reference for positioning the cup in THA.
6	Abe (2012)^10^	80 (38/42)	Using 3D reconstruction CT measuring and comparing between 2 groups ON and OA when using TAL as anatomy landmark intraoperative	In ON hips, TAL is a good guide for determining cup orientation during THA.
7	Miyoshi (2012)^11^	104 (12/92) with 114 hips	Using TAL as landmark during THA and evaluated post-op by CT scanner measured the orientation of cup acetabula	The TAL is a practical anatomy landmark for determining cup anteversion in THA.
8	Inoue (2013)^12^	29 (10/19) with 31 hips	Using TAL landmark when THA for 3 groups: OA, ON secondary to DDH, and congenital hip dislocation.	The TAL is a useful anatomic landmark for THA in dislocated hips.
9	Jain (2013)^13^	NA	A systematic literature review to determine TAL intraoperative and using TAL to orient anteversion cup in THA	That was good evidence for the use the TAL in terms of the accuracy of acetabular component.
10	Meermans (2014)^14^	80 (NA)	Comparative between 2 group: free hand and TAL	The use TAL in determining anteversion has led to reduction in the rate of dislocation. The advantage of using this landmark is that it is independent of position of the patient on the operating table.
11	Hiddema (2016)^15^	9 (NA) with 16 hips	Cadavers study using navigation system measured inclination and version of the acetabular component in 3 positions with low edge of cup: flush with; 5 mm proximal to; 5 mm distal to border of TAL	Cup inclination was acceptable when lower edge of the cup was flush or within 5 mm proximal to the TAL.
12	El Idrissi (2016)^16^	21 (NA)	Using TAL landmark and evaluated post-op on Xray measured inclination and anteversion of cup	The TAL can assist in positioning the acetabular component version without need for an external instrument and independent of the patient’s position.
13	Revanngowda (2017)^3^	51 (35/16) with 52 hips	Using TAL landmark and evaluated post-op on Xray measured inclination and anteversion of cup	82.6% anteversion of cup and 76.9% inclination of cup within LSZ.
14	Agarwal (2020)^17^	40 (NA)	Comparative between 2 groups: using TAL and mechanic alignment guide.	TAL is patient specific intraoperative landmark which not affected by patient positioning while angle guide device can give false positive assessment of cup version.
15	Pazhani (2020)^18^	35 (NA)	Using TAL for determing inclination cup component by measuring distance from inferior rim the cup to the TAL. Assessment post-op inclination on xray	TAL can be identified in all case can be used as a denpenable intraoperative landmark for maintaining the cup inclination when the cup positioned at a depth less than 8 mm from the TAL.
16	Li Long (2021)^19^	192 (84/108) with 384 hips	Study with hip joint specimens. CT-scanner and measuring anteversion of TAL and acetabulum	There was a significant correlation between TAL anteversion and acetabular anteversion. TAL can be used as an anatomical marker for locating acetabular cup anteversion in THA.
17	Ning (2023)^20^	19 studies in 3274 patients (NA) with 3550 hips	Systematic literature review using keywords THA, THR, total hip prosthesis, TAL	TAL can reliably be used to align the acetabular component in the safe zone for anteversion and inclination in THA.
18	Singh (2024)^21^	30 (NA)	Using TAL to determine both anteversion and inclination of cup. CT-3D post-op evaluated orientation.	TAL is a reliable, inexpensive and patient-specific intraoperative anatomical landmark for the placement of acetabular components.
19	Our study (2024)	109 (84/38) with 122 hips	Pre-op measured orientation anteversion and inlination of TAL and acetabulum on CT-MPR and MR-a. assessment correlation with orientation of TAL and acetabulum intraoperative	There was significant correlation between orientation TAL and acetabulum on CT-MPR, MR-a and intraoperative.

Our study demonstrates a positive correlation in the orientation of the TAL and acetabulum across CT-MPR, MR-a, and intraoperative measurements. Thereby, we recommend that the TAL be used as a patient-specific anatomical landmark to orient the acetabulum when positioning it parallel to the TAL. However, we acknowledge that the TAL may not always be well defined in all cases, which could affect its utility as an anatomical reference. In cases where the TAL is less visible or obscured due to anatomical variations or pathologies, it may be challenging to use it as a reliable landmark. Surgeons should consider alternative anatomical landmarks or imaging guidance in such situations.

Additionally, we recognize that preoperative CT assessment of acetabular anteversion could be influenced by osteophytes, which may obscure the natural acetabular contours, potentially altering the measurements. In this study, measurements were carefully evaluated by experienced radiologists to minimize this impact, but this does not completely eliminate inaccuracies. Furthermore, interobserver variability remains a valid concern despite the use of standardized protocols. Future research should include quantitative analyses of measurement errors and strategies to address these challenges, such as advanced imaging algorithms or automated measurement systems.

Additionally, while preoperative imaging, including CT and MRI, is not routinely used specifically to assess TAL orientation in current practice, it may be valuable for precise preoperative planning, especially in patients with challenging anatomy or those at risk for complications such as dislocation. In our study, the preoperative imaging techniques were applied specifically for research purposes, providing detailed insights into the correlation between TAL orientation and acetabular positioning. Although TAL alignment is not a standard preoperative procedure in routine clinical practice, incorporating it into the planning phase could enhance the accuracy of acetabular component placement, particularly in complex cases.

Also herein, this study presents certain limitations. First, standardized procedures for measuring hip joint and acetabular parameters in Vietnamese patients are lacking; thus, the techniques used in this study were developed and implemented for the first time by the research team at E Hospital. Second, the reliability and validity of the handmade protractor remain unverified, and its inability to account for intraoperative pelvic motion, a critical factor in acetabular alignment, limits its accuracy. Future iterations should incorporate technologies like real-time pelvic tracking to address this issue. Additionally, a quantitative analysis of measurement errors was not performed, and the protractor’s design, tailored to Vietnamese anatomy, may limit its applicability to other populations. Furthermore, future studies should include a preliminary validation of the protractor by comparing its measurements to those obtained using established electronic navigation systems, to further evaluate its accuracy and applicability in clinical practice. Lastly, while significant correlations were observed between intraoperative and imaging-based measurements, the study did not assess long-term clinical outcomes such as dislocation or impingement rates. Two cases of postoperative dislocation were observed during the follow-up period. Both patients had acetabular components positioned within Lewinnek’s “safe zone” as confirmed by postoperative imaging. One patient experienced a domestic slip-and-fall accident, while the other was involved in a motorbike accident. Both dislocations were managed with closed reduction under spinal anesthesia, and no further dislocations were observed during the 12-month follow-up period. These cases highlight the importance of external factors, such as trauma, in contributing to dislocation risk, even when optimal acetabular positioning is achieved. These limitations underscore the need for further research to validate the device and explore the clinical efficacy of TAL-guided cup positioning in diverse populations.

## Conclusions

The TAL is a personal, visible, and useful landmark for anatomical alignment in acetabular cup positioning during total hip replacement. Utilizing this anatomical landmark can help position the cup within the Lewinnek’s safe zone and reduce the incidence of postoperative dislocation and impingement.

## Data Availability

The data used in this article are available upon request from the corresponding author. The data are not publicly available due to the protection of personal data.
